# Tumor-targeting adenovirus OBP-401 inhibits primary and metastatic tumor growth of triple-negative breast cancer in orthotopic nude-mouse models

**DOI:** 10.18632/oncotarget.13296

**Published:** 2016-11-11

**Authors:** Shuya Yano, Kiyoto Takehara, Hiroyuki Kishimoto, Hiroshi Tazawa, Yasuo Urata, Shunsuke Kagawa, Michael Bouvet, Toshiyoshi Fujiwara, Robert M. Hoffman

**Affiliations:** ^1^ AntiCancer, Inc., San Diego, CA, USA; ^2^ Department of Surgery, University of California San Diego, CA, USA; ^3^ Department of Gastroenterological Surgery, Okayama University, Graduate School of Medicine, Dentistry and Pharmaceutical Sciences, Okayama, Japan; ^4^ Center for Innovative Clinical Medicine, Okayama University Hospital, Okayama, Japan; ^5^ Oncolys BioPharm Inc., Tokyo, Japan

**Keywords:** triple-negative breast cancer, TNBC, high-metastatic, variants, nude mouse, adenovirus, OBP-401

## Abstract

Our laboratory previously developed a highly-invasive, triple-negative breast cancer (TNBC) variant using serial orthotopic implantation of the human MDA-MB-231 cell line in nude mice. The isolated variant was highly-invasive in the mammary gland and lymphatic channels and metastasized to lymph nodes in 10 of 12 mice compared to 2 of 12 of the parental cell line. In the present study, the tumor-selective telomerase dependent OBP-401 adenovirus was injected intratumorally (i.t.) (1 × 10^8^ PFU) when the high-metastatic MDA-MB-231 primary tumor expressing red fluorescent protein (MDA-MB-231-RFP) reached approximately 500 mm^3^ (diameter; 10 mm). The mock-infected orthotopic primary tumor grew rapidly. After i.t. OBP-401 injection, the growth of the orthotopic tumors was arrested. Six weeks after implantation, the fluorescent area and fluorescence intensity showed no increase from the beginning of treatment. OBP-401 was then injected into high-metastatic MDA-MB-231-RFP primary orthotopic tumor growing in mice which already had developed metastasis within lymphatic ducts. All 7 of 7 control mice subsequently developed lymph node metastasis. In contrast, none of 7 mice which received OBP-401 had lymph node metastasis. Seven of 7 control mice also had gross lung metastasis. In contrast, none of the 7 mice which received OBP-401 had gross lung metastasis. Confocal laser microscopy imaging demonstrated that all control mice had diffuse lung metastases. In contrast, all 7 mice which received OBP-401 only had a few metastatic cells in the lung. OBP-401 treatment significantly extended survival of the treated mice.

## INTRODUCTION

Triple-negative breast cancer (TNBC) lack estrogen, progesterone, and ErbB2 receptors, and is almost always resistant to therapy [1, 2]

We previously described the development of a highly-invasive, triple-negative breast cancer (TNBC) variant using serial orthotopic implantation of MDA-MB-231 human breast cancer in nude mice. The isolated variant was highly invasive in the mammary gland and metastasized to lymph nodes in 10 of 12 mice compared to 2 of 12 of the parental cell line [3].

Surgical resection of the MDA-MB-231 high-metastatic variant primary tumor in orthotopic models resulted in rapid and enhanced lymphatic trafficking of residual cancer cells and subsequent extensive lymph-node and lung metastasis that did not occur in the non-surgical mice [4].

The parental MDA-MB-231 grew as spindle-shaped cells on plastic dishes. In contrast, highly-metastatic MDA-MB-231 cells, derived from lymph node metastasis, grew mostly in suspension suggesting that high-metastatic MDA-MB-231 became undifferentiated, therefore more aggressive and metastatic. After wounding confluent co-cultures on plastic, the high-metastatic variant expressing red fluorescent protein MDA-MB-231-RFP cells significantly migrated and invaded more extensively compared with low-metastatic parental MDA-MB-231 expressing green fluorescent protein (MDA-MB-231-GFP) cells, as imaged by color-coded confocal microscopy [5].

We have previously developed a genetically-engineered GFP-expressing telomerase-specific adenovirus, OBP-401, which can selectively illuminate and kill cancer cells [6–14].

We previously demonstrated that targeting human cancer in nude mice with OBP-401 enabled effective fluorescence-guided surgery (FGS) of many tumor types in orthotopic models [6–14]. OBP-401 enabled complete resection and prevented local recurrence and greatly inhibited lymph node metastasis of the high-metastatic MDA-MB-231 variant TNBC [15].

In the present report we demonstrate that OBP-401 alone can inhibit primary-tumor growth and metastasis of the high-metastatic MDA-MD-231 variant.

## RESULTS AND DISCUSSION

### Efficacy of OBP-401 against high-metastatic MDA-MB-RFP orthotopic primary tumors

**Figure 1:**
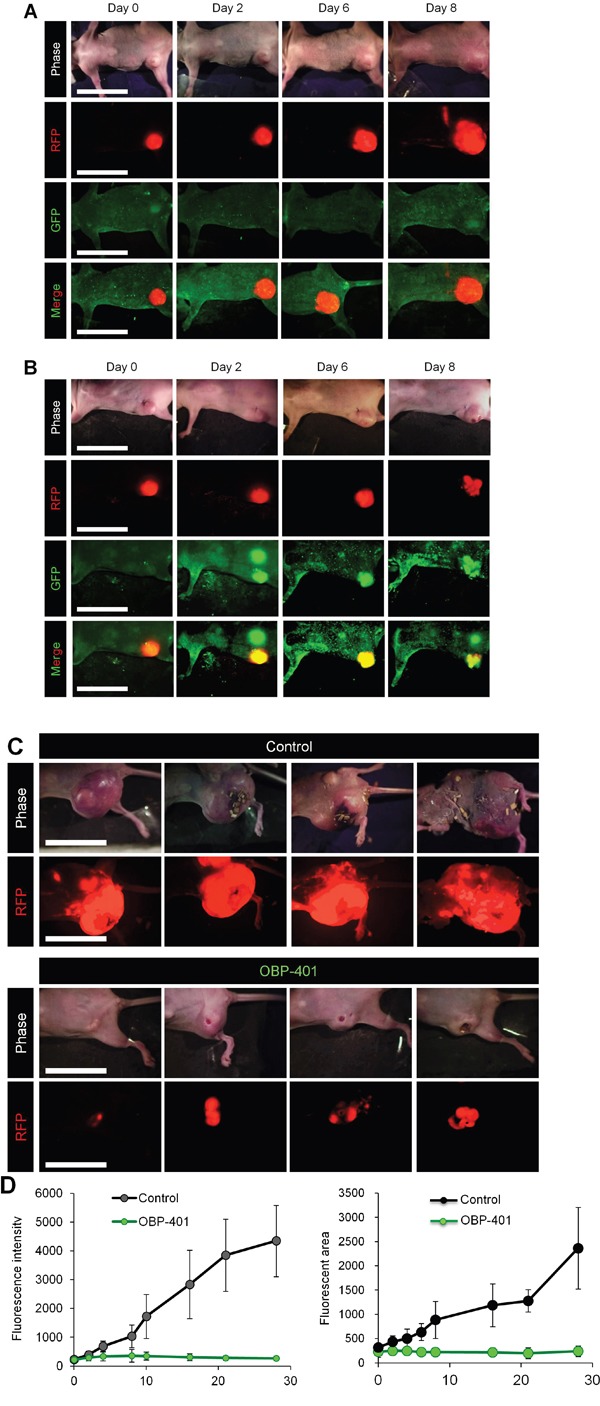
Efficacy of OBP-401 against primary orthotopic high-metastatic MDA-MB-231-RFP OBP-401 was injected intratumorally (1 × 10^8^ PFU) when primary tumors reached approximately 500 mm^3^ (diameter; 10 mm). **A.** Representative whole-body images of mock-infected orthotopic primary tumor. **B.** Representative whole-body images of orthotopic tumors before and after injection of OBP-401. **C.** Representative whole-body images of mock-infected and OBP-401-treated orthotopic tumors 6 weeks after tumor implantation. **D.** Comparison of primary tumor growth of mock-infected and OBP-401-treated mice. Fluorescent area and fluorescence intensity are calculated with ImageJ software. Data are shown as average ± SD. N = 7.

OBP-401 was injected intratumorally (1 × 10^8^ PFU) when high-metastatic MDA-MB-231-RFP primary tumors reached approximately 500 mm^3^ (diameter; 10 mm) (Figure 1). The mock-infected orthotopic primary tumor grew rapidly. After intra-tumoral (i.t.) OBP-401 injection, the growth of the orthotopic primary tumors was arrested. Six weeks after implantation, the fluorescent area and fluorescence intensity of the OBP-401-treated tumors showed no increase from the beginning of treatment.

### Efficacy of OBP-401 against high-metastatic MBA-MB-231 lymphatic trafficking and metastasis

**Figure 2:**
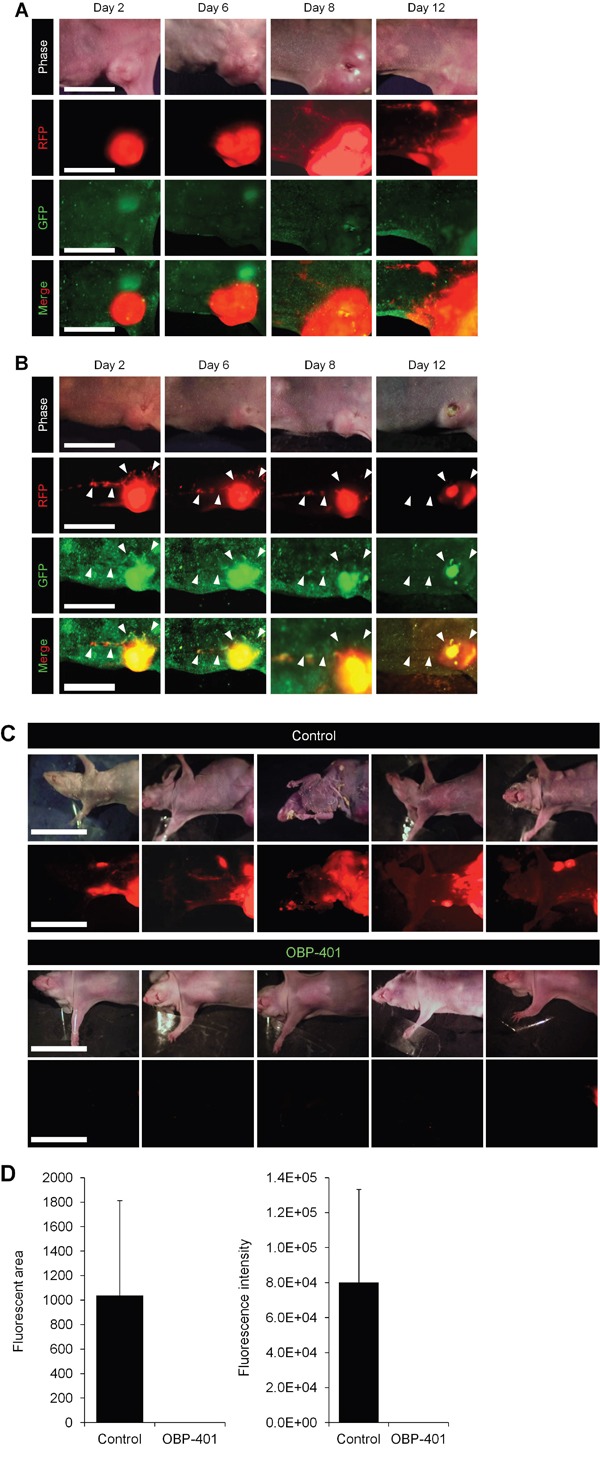
Efficacy of OBP-401 against lymph-node metastasis of high-metastatic MDA-MB-231-RFP OBP-401 was injected intratumorally (1 × 10^8^ PFU) when the mice already developed metastasis in lymphatic ducts. **A.** Representative whole-body time-course images of mock-infected orthotopic tumors. **B.** Representative whole-body time-course images of orthotopic tumors after injection of OBP-401. **C.** Representative whole-body images of mock-infected and OBP-401-treated mice 6 weeks after tumor implantation. **D.** Comparison of lymphatic metastasis of mock-infected and OBP-401-treated mice. Fluorescent area and fluorescence intensity of lymph-node metastasis are calculated with ImageJ software. Data are shown as average ± SD. N = 7.

OBP-401 was injected into high-metastatic MDA-MB-231-RFP primary tumors growing in mice which had already developed metastasis in lymphatic ducts (Figure 2A). OBP-401 labeled metastatic cancer cells with GFP in lymphatic ducts, as well as the primary tumors by 3 days after injection (Figure 2B). Intravital real-time GFP imaging demonstrated that metastatic cancer cells were killed after 3 cycles of OBP-401 treatment (Figure 2B). All 7 of 7 control mice had lymph node metastasis, 6 weeks after orthotopic implantation (Figure 2C). In contrast, none of 7 mice which were treated with OBP-401 had lymph-node metastasis (Figure 2C).

### OBP-401 inhibits lung metastasis of high-metastatic MDA-MB-231 RFP

**Figure 3:**
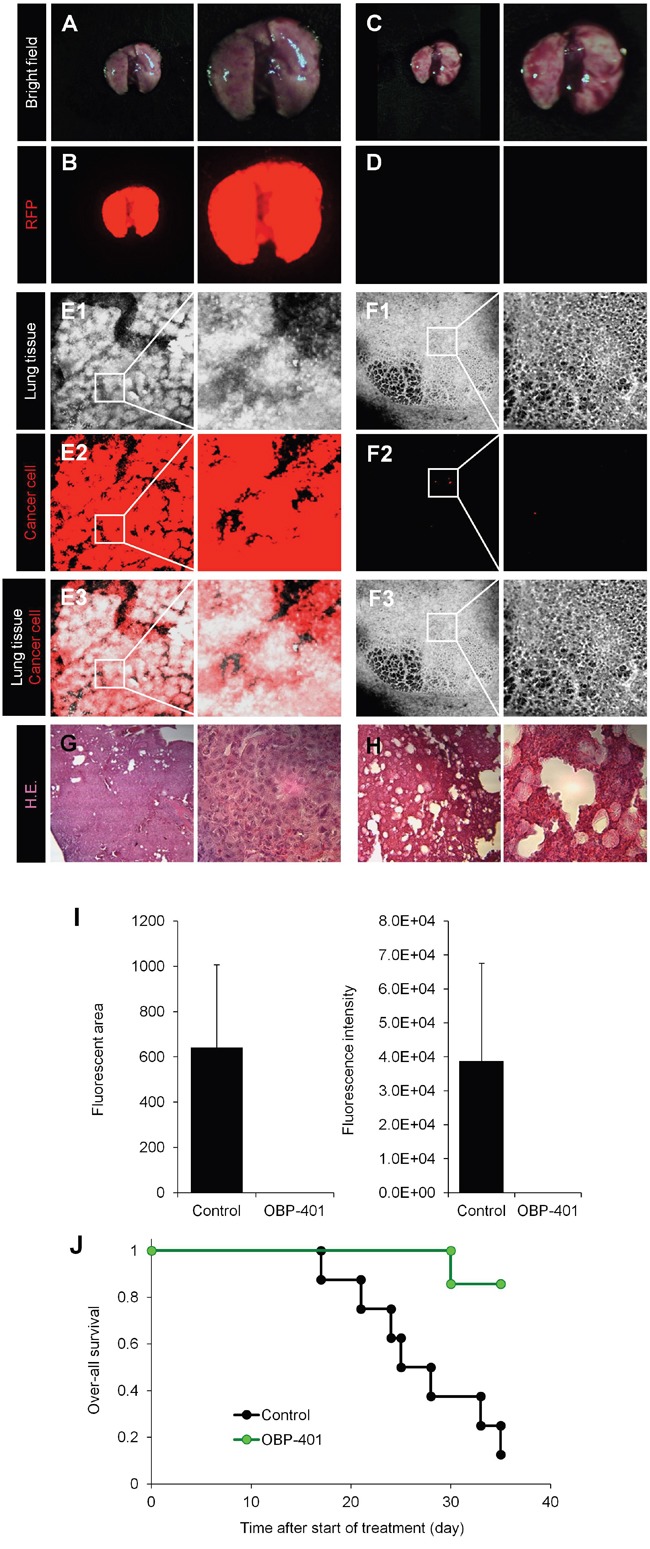
Efficacy of OBP-401 on lung metastasis of high-metastatic MDB-MB-231-RFP and survival **A-D.** Representative whole-lung images of mock-infected (A, B) and OBP-401-treated mice (C, D). A, C: Bright-field; B, D: fluorescence images. Images were acquired with the OV100 whole-body imaging system (Olympus, Japan). **E** and **F.** Representative single-cell images of lung in mock-infected (E) and OBP-401-treated mice (F). E1, F1: Lung tissue imaged by autofluorescence; E2: F2; fluorescence images. E3, F3; Composite images from E1, F1 and E2, F2. **G** and **H.** Representative H&E. images of lung in mock-infected (G) and OBP-401-treated mice (H). **I.** Comparison of lung metastasis of mock and OBP-401-treated mice. Fluorescent area (left) and fluorescence intensity (right) of lung metastasis are calculated with ImageJ software. Data are shown as average ± SD. N = 7. **J.** Kaplan-Meyer shows the over-all survival of control and OBP-401.

Seven of 7 control mice had gross lung metastasis six weeks after orthothopic implantation (Figure 3A and 3B). In contrast, none of the 7 mice which were treated with OBP-401 had gross lung metastasis (Figure 3C and 3D). Confocal laser microscopy imaging demonstrated that all control mice had diffuse lung metastases (Figure 3E, 3G). In contrast, the 7 mice which were treated with OBP-401 had only a few metastatic cells in the lung (Figure 3F, 3H). The fluorescent area and fluorescence intensity in the lungs was greatly reduced by OBP-401 (Figure 3I).

OBP-401 treatment significantly extended survival of the treated mice (Figure 3J). Thus, injection of OBP-401 into the primary tumor inhibited local and distant metastasis and prolonged the survival rate.

TNBC is a recalcitrant disease with few effective treatment options in the clinic [1, 2]. Although standard chemotherapy drugs have had some effect with claims of “profound prevention of experimental brain metastasis” of breast cancer [16], such treatment is of limited efficacy.

OBP-401 appears to be highly effective against primary growth, lymph-node metastasis and lung metastasis of a high-metastatic variant of TNBC. The parent of OBP-401, OBP-301, has already undergone clinical trials demonstrating safety [17]. Clinical development of tumor-targeting OBP-401 is warranted.

OBP-401 will be tested on breast cancer patient-derived orthotopic xenografts (PDOX) [18, 19] in the next set of studies.

Previously-developed concepts and strategies of highly selective tumor targeting can take advantage of molecular targeting of tumors, including tissue-selective therapy which focuses on unique differences between normal and tumor tissues [20–25].

## MATERIALS AND METHODS

### OBP-401

Telomerase-dependent adenovirus OBP-401 contains a promoter element of the human telomerase reverse transcriptase (*hTERT*) gene which drives the expression of E1A and E1B genes linked to an internal ribosome entry site for selective replication only in cancer cells. The GFP gene of the virus is driven by the CMV promoter, was constructed as previously described [6–14].

### Cell culture

Parental and high-metastatic variants of MDA-MB-231P-RFP were maintained and cultured in DMEM medium with 10% fetal bovine serum (FBS) and 5% penicillin/streptomycin [5].

### Mice

Athymic nude mice (AntiCancer, Inc., San Diego, CA) were kept in a barrier facility under HEPA filtration. Mice were fed with autoclaved laboratory rodent diet (Tecklad LM-485, Western Research Products). All animal studies were conducted in accordance with the principals and procedures outlined in the National Institutes of Health Guide for the Care and Use of Laboratory Animals under assurance A3873– 01.

### Establishment of orthotopic, highly metastatic breast cancer

Initially, MDA-MB-231-RFP cells (1 × 10^7^ cells/site) were injected subcutaneously in the flank of nude mice. For orthotopic transplantation, fragments of the harvested subcutaneous tumor were grafted in a mammary gland of nude mice. After growth, the tumor was resected. Residual cancer cells grew into a tumor which was harvested and divided into fragments and re-implanted into a mammary gland of nude mice. After the tumor metastasized to lymph nodes, it was harvested and divided into fragments and re-implanted into mammary gland of nude mice. After growth, the orthotopic tumor was resected. The residual cancer cells formed primary and metastatic tumors. The metastasis was harvested, divided into fragments and re-implanted into the mammary gland of nude mice. Highly-metastatic MDA-MB-231 tumors were developed after seven orthotopic transplantations described above [3].

### OBP-401 treatment

OBP-401 (1 × 10^8^ PFU) was intratumorally injected into the orthotopic tumor every 3 days.

### *In vivo* whole-body/whole-tumor Imaging

For whole-body or whole-tumor imaging, an Olympus small animal imaging system, OV-100, was used. The OV100 small animal imaging system (Olympus Corp., Tokyo, Japan), was used. The OV100 contains an MT-20 light source (Olympus Biosystems, Planegg, Germany) and DP70 CCD camera (Olympus), for whole body, as well as subcellular imaging in live mice [26–29]. The optics of the OV100 have been specially developed for macroimaging as well as microimaging with high light-gathering capacity. Four individually optimized objective lenses, parcentered and parfocal, provide a 105-fold magnification range. High-resolution images were captured directly on a PC (Fujitsu Siemens, Munich, Germany). Images were processed for contrast and brightness and analyzed with the use of Paint Shop Pro 8 and CellR [30].

### Statistical analysis

Data are shown as means ± standard deviation (SD). For comparison between two groups, significant differences were determined using the Student's t-test. *P* values of < 0.05 were considered significant. Pearson chi-square analysis was used to compare the rate of lymph node metastasis between control and OBP-401-treated groups. Statistical analysis for over-all survival was performed using the Kaplan-Meier test along with log-rank test.

## Abbreviations

GFP: green fluorescent protein
RFP: red fluorescent protein
